# Do Low Hemoglobin Levels Affect the Healing Process of Periprosthetic Joint Infection?

**DOI:** 10.7759/cureus.14393

**Published:** 2021-04-09

**Authors:** Yüksel Uğur Yaradılmış, Ahmet Ateş, Mehmet Özer, Erdi Özdemir, İsmail Demirkale, Murat Altay

**Affiliations:** 1 Orthopaedics and Traumatology, Keçiören Health Practice and Research Center, Ankara, TUR

**Keywords:** periprosthetic joint infection, hemoglobin, transfusion, revision knee arthroplasty, two-stage surgery

## Abstract

Background

Revision knee arthroplasty (RKA) is associated with low hemoglobin (Hb) levels after surgery, which results mostly from perioperative blood loss. Periprosthetic joint infection (PJI) is one of the common reasons for RKA. This study aimed to determine whether low Hb levels affect the healing process of PJI.

Methodology

This retrospective study included 69 patients who underwent two-stage revision for PJI between 2013 and 2016. Patients were divided into two groups according to the latest Hb levels (Hb < 10 and Hb > 10 g/dL) during hospitalization for the first-stage revision surgery. Laboratory parameters of infection were measured during the cement spacer retention period: C-reactive protein (CRP), sedimentation rate (SEDIM), and white blood cell (WBC) count. Treatment was evaluated in two periods: cement spacer retention period (between the first surgery and second surgery) and the first normal CRP period (between the first surgery with the first normal CRP level during the cement spacer retention period). Infection parameters in the two time periods and reoperation with cement spacer were compared between the groups.

Results

The mean patient age was 67.3 ± 7.94 (50-87) years, and the female-to-male ratio was 4:1. No difference was found in the postoperative first control CRP, SEDIM, and WBC between the groups (p = 0.953, p = 0.3341, and p = 0.444, respectively). CRP-SEDIM control curves were observed in parallel, and no significant difference was found. The cement spacer retention period was 60.3 ± 24.8 (17-123) days, and the first normal CRP period was 87.3 ± 28.4 (14-161) days; no statistical difference was found between the groups (p = 0.727, p = 0.754).

Conclusions

In RKA, as low Hb level was not a negative factor of infection, blood transfusion should be avoided as it has many complications.

## Introduction

Periprosthetic joint infection (PJI) is the major reason for revision knee arthroplasty (RKA), accounting for 25% of all RKA cases [1]. Aside from being the main complication, it ranks the highest in the dissatisfaction levels in patients [2].

In PJI, two main treatment modalities are identified, i.e., single-stage revision and two-stage revision. The two-stage RKA has much better outcomes, and it is known as the gold standard treatment [3,4]. Two-stage RKA includes the use of antibiotic-impregnated cement spacer retention period that lasts from placing the cement spacer into the knee to removing it just before re-implantation.

Total knee arthroplasty (TKA) and RKA were associated with low hemoglobin (Hb) levels after surgery, which results mostly from perioperative blood loss. RKA was considered to result in more severe blood loss than TKA [5]. As internal medicine and orthopedic surgeons suggest, blood transfusion is performed if Hb < 8 g/dL [6]. However, low Hb levels postoperatively may be an obstacle in surgical wound healing. In primary TKA cases, Namba et al. reported that lower postoperative Hb levels could be a risk factor for PJI [7]. However, low Hb levels after the cement spacer retention period might play a role in the healing process.

Blood transfusion is not above suspicion because it has fatal complications, including multiple organ dysfunctions, pneumonia, perioperative immunosuppression, or postoperative immunosuppression [8]. Reducing blood loss volume and blood transfusion rate is crucial for patients undergoing TKA and RKA. Thus, this study aimed to determine whether lower Hb affects the healing process of PJI.

## Materials and methods

Patients and study design

This retrospective study included 206 patients undergoing RKA between 2013 and 2019. This study was conducted in the Keçiören Health Practice and Research Hospital, and ethical approval was obtained from the same hospital (Project No: 43278876, Date: 28/12/2020). Patients with non-septic RKA, single-stage septic revision, and missing data and who did not provide consent for study participation were excluded. Finally, the study included 69 patients who underwent two-stage septic revision surgery for PJI. PJI was diagnosed using the Musculoskeletal Infection Society criteria, including clinical finding and laboratory results [9].

**Figure 1:**
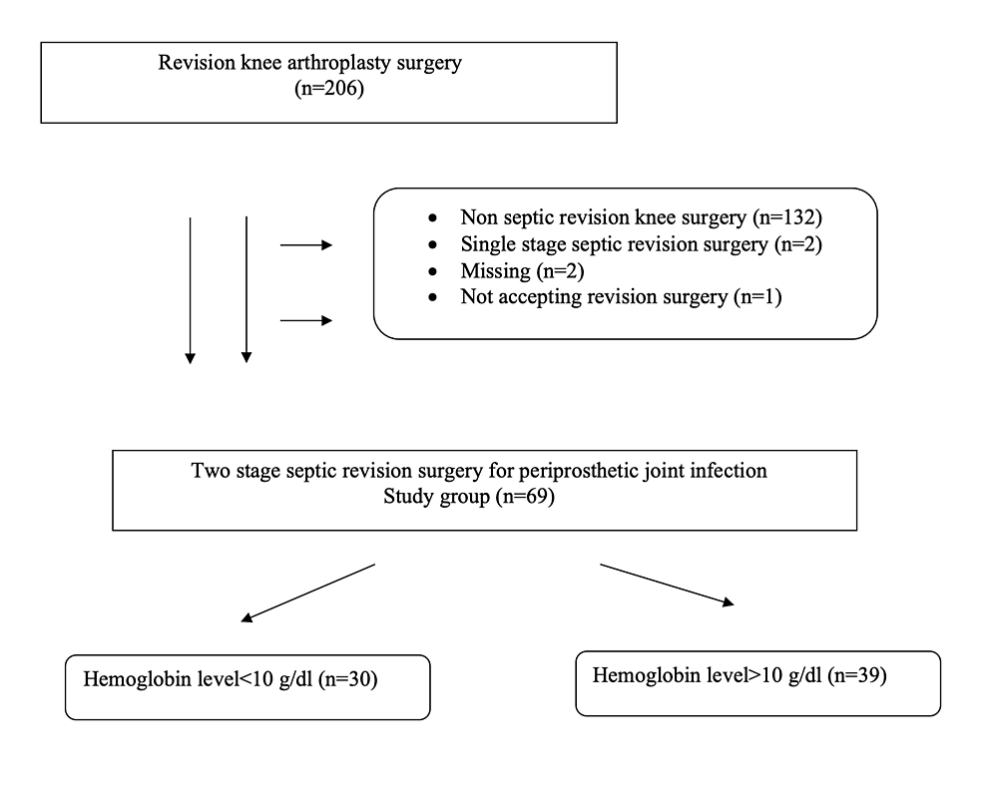
Flow chart of patients.

Demographic data and examination results of the patients were recorded. The dates of the first surgery (cement spacer implantation), first normal C-reactive protein (CRP), and second surgery (re-implantation) were recorded. Cement spacer retention time was determined between the first surgery and the second surgery. Patients’ laboratory evaluations including Hb, acute-phase reactants such as CRP, and sedimentation rate (SEDIM) were measured preoperatively, postoperative day one, and before discharge during the cement spacer retention period. Patients were grouped according to the final Hb values before discharge during the first surgery: Hb < 10 g/dL (group 1) and Hb > 10 g/dL (group 2) (Figure 1). Infection parameters at the two time period were compared by groups. Evaluation of infection treatment (infection parameters, two time periods, and re-implantation with cement spacer), operation time, postoperative blood transfusion, re-hospitalizations, and final the Western Ontario and McMaster Universities Osteoarthritis Index (WOMAC) score were compared by groups.

Surgical procedures

The first stage of surgery was carried out by removing prosthesis and implantation of an antibiotic-impregnated cemented spacer. The procedure was performed in the supine position. The medial parapatellar approach was used in all cases, and the quadriceps snip procedure was added if needed. None of the patients needed tuberosity osteotomy. For each patient, five intra-articular cultures were taken perioperatively. After prosthesis removal, irrigation was performed with low-pressure lavage. A customized or readily available cement spacer was applied. One packet of antibiotic-loaded cement was used additionally for surface suitability while applying the prepared cement. Customized cements were shaped using two packets of antibiotic cements. All spacer implantations were movable and contained gentamicin. The cement was irrigated again after cooling.

On day one after the first surgery, the patients were mobilized with a walker. Weight-bearing was permitted as tolerated with a brace. Blood transfusion was performed to patients who manifest symptoms and had Hb < 8. Postoperative empirical antibiotic treatment was initiated. Ciprofloxacin and teicoplanin were used as empirical antibiotics therapy for all patients. Once culture results were available, acute-phase reactant samples were taken again, and antibiotic treatment was arranged by the infection clinic before the patient was discharged. The patients were called for follow-up examinations at two-week intervals, and Hb, CRP, and SEDIM levels were monitored and controlled. During follow-up, when the CRP values were normal, the second stage of the surgery was applied with a two-week window period to maintain normal CRP values without antibiotic usage. Cement spacer reoperation was planned for patients whose CRP values do not return to normal within three months.

When no signs of infection were present, patients were prepared for the second surgery (re-implantation). The patients were followed up every three months in the first year and yearly thereafter.

Evaluation of infection

Laboratory infection parameters (WBC, CRP, and SEDIM values) were compared between the groups during the cement spacer retention period. Infection was also evaluated during the cement spacer retention period (between the first surgery and second surgery) and the first normal CRP period (between the first surgery with the first normal CRP level on the cement spacer retention period). Parameters measured in these two periods were compared and evaluated by groups. In addition, patients who did not show a decrease in CRP and had reoperation with a cement spacer were identified, and they were compared by groups.

Statistical analysis

Collected data were analyzed using SPSS version 22 software (IBM Corp., Armonk, NY, USA) and at a confidence interval of 95%. Qualitative data were stated as frequency distribution, and quantitative data were presented as mean, minimum, and maximum values. The Chi-square test was applied to analyze categorical data, and Student’s t-test was used to evaluate quantitative data. Normality of data distribution was tested using the Shapiro-Wilk test. Inter-observer and intra-observer reliabilities were assessed using the interclass correlation coefficient. Postoperative CRP, SEDIM, WBC values, operation time, postoperative blood transfusion, postoperative hospitalizations, and WOMAC scores were compared with the Mann-Whitney U-test. The cement spacer retention period and the first normal CRP period showed correlation during the Spearman’s correlation test. The time interval of the cement spacer retention and first normal CRP period were compared using the Mann-Whitney U-test. Data of patients who did not show a decrease in CRP level and had reoperation with cement spacer were compared using the Chi-square test. A value of p < 0.05 was accepted as significant.

## Results

The mean age of the patients was 67.3 ± 7.94 (range, 65-93) years, and the female-to-male ratio was 4:1. The mean follow-up duration was 36 ± 6.7 (range, 74-12) months. The comparison of group data is shown in Table 1.

**Table 1:** Demographic data of the groups. Hb, hemoglobin

	Hb < 10 g/dL	Hb > 10 g/dL	Total	P-Value
Patients	30	39	69	
Hb (mean)	9.28 ± 0.42 (8–9.9)	10.37 ± 0.46 (10–11.3)		0.000
Age (years)	65.4 ± 7.6	68.5 ± 8.9	67.3 ± 7.94	0.182
Gender (female/male)	24/6	29/10	(53/16)	0.235
Side (right/left)	15/15	21/18	36/33	0.562
Follow-up (months)	36 ± 7.1 (72–12)	34 ± 6.3 (74–12)	38 ± 6.8 (74–12)	0.764

No difference was found in the preoperative and postoperative first control CRP, SEDIM, and WBC between the groups (Table 2).

**Table 2:** Comparisons of laboratory infection parameters between the groups. Hb, hemoglobin; CRP, C-reactive protein; SEDIM, sedimentation rate; WBC, white blood cell

	Hb < 10 g/dL	Hb > 10 g/dL	P-Value
Preoperative			
CRP	49.6 ± 33(13–124)	43.2 ± 31.9 (6.7–157)	0.494
SEDIM	70.7 ± 29.4 (19–126)	62.3 ± 24.9 (25–120)	0.248
WBC	7.6 ± 2.2 (3–14.2)	7.9 ± 2.5 (4–13.8)	0.941
Postoperative			
CRP	96.8 ± 61 (12–272)	97.47 ± 58.8 (10.6–243)	0.953
SEDIM	78.3 ± 26.2 (24–120)	68.75 ± 32 (11–122)	0.341
WBC	7.6 ± 26 (4.6–16.7)	7.8 ± 1.8 (4.5–13.1)	0.449

**Figure 2:**
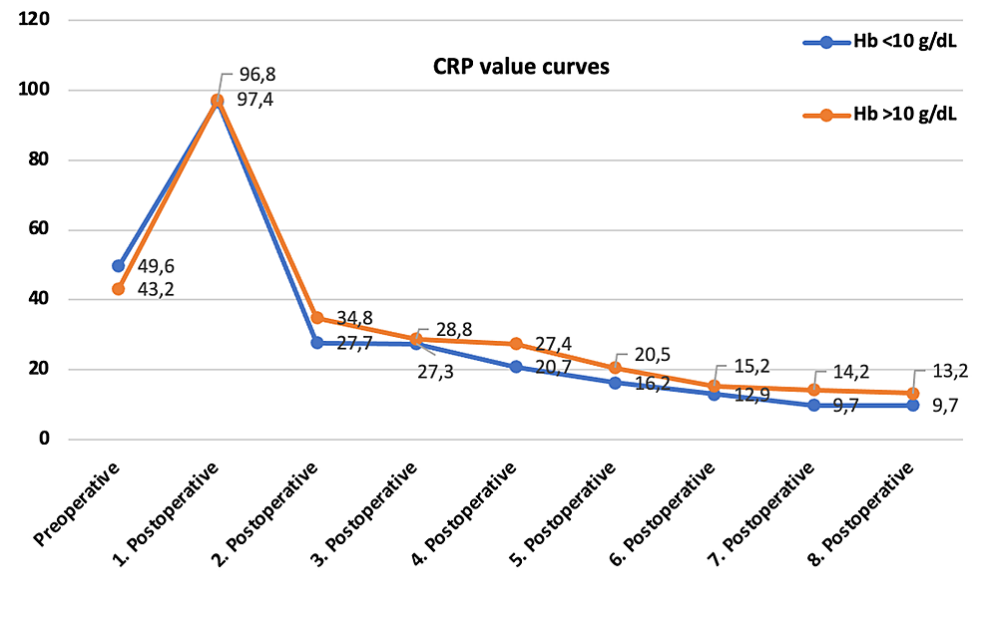
CRP value changes in follow-ups by groups. CRP, C-reactive protein

**Figure 3:**
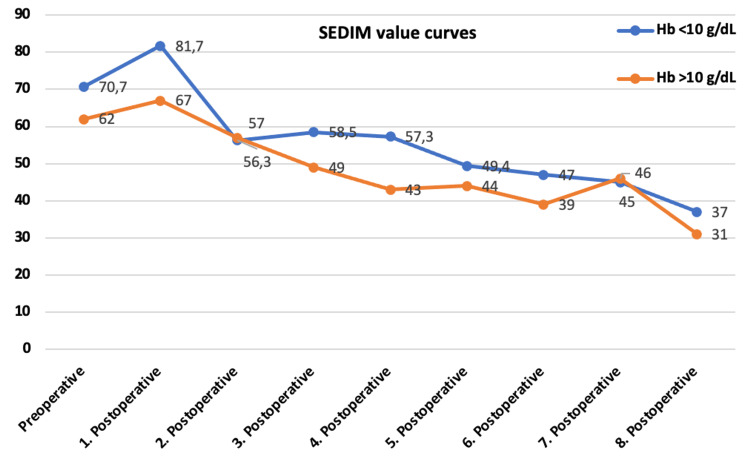
SEDIM value changes in follow-ups by groups. SEDIM, sedimentation rate

CRP and SEDIM control curves were parallel, and no statistical difference was found (Figures 2 and 3).

The cement spacer retention period was 87.3 ± 28.4 (14-161) days, and the first normal CRP period was 60.3 ± 24.8 (17-123) days; no significant difference was found between the groups (p = 0.727, p = 0.754).

During follow-up, CRP levels increased in six patients for three months, and no significant difference was observed in each group (p = 0.340). Four patients underwent cement spacer re-implantation because specimens were suspected to be frozen, and two patients underwent second surgery because they have normal frozen specimens. No difference was found in terms of operation time, postoperative blood transfusions, and re-hospitalizations (p = 0.860, p = 0.880, p = 0.760, respectively).

After the second stage surgery, two patients showed signs of infection, so debridement and irrigation were performed. Infection signs were not observed in 67 patients. The final WOMAC score was 75 ± 14, with no difference between the groups (p = 0.797) (Table 3).

**Table 3:** Comparisons of clinical results between the groups. Hb, hemoglobin; CRP, C-reactive protein; WOMAC, Western Ontario and McMaster Universities Osteoarthritis Index

	Hb < 10 g/dL	Hb > 10 g/dL	P-Value
Cement spacer retention period	87.5 ± 33.6 (14–161)	87.1 ± 23.9 (40–156)	0.727
First normal CRP period	60.6 ± 27.7 (17–123)	60.18 ± 22.7 (19–99)	0.754
No decrease in CRP level	3	3	0.920
Reoperation with cement spacer	1	3	0.230
Surgery time (minutes)	65.3 ± 11	61.2 ± 12	0.860
Blood transfusion (units)	1.2 ± 0.2	1.1 ± 0.2	0.880
Duration of hospitalization after surgery (days)	5.5 ± 1.2	5.4 ± 1.1	0.760
WOMAC score (after revision arthroplasty)	75	76	0.797

## Discussion

This study was carried out to examine whether low Hb levels affect the healing process of PJI. This study shows that low Hb levels (Hb > 8 g/dL) is not a negative parameter in the curability of PJI.

Although the use of tourniquet is controversial, TKA is carried out with tourniquet in our clinic [10,11]. Tahmasebi et al. reported that approximately 265 mL (intraoperatively) and 554 mL (postoperatively) of blood loss is expected in such major surgery [12]. Parvizi et al. revealed that PJI occurs more often in patients with anemia (4.3%) than in patients without anemia (2%) [13]. Many studies have associated anemia with increased risk of infection, length of hospital stay, and mortality in patients who underwent surgery [14-16]. Prased et al. reported that TKA is associated with lesser blood loss than RKA [5]. Pruzansky et al. assumed that the TKA is the second most common modifiable risk factor, while the most common risk factor is RKA [16]. According to Frisch et al., the incidence of deep infection after TKA was higher in the transfusion group than in the non-transfusion group (2.4% vs. 5%) [17]. As RKA involves extensive soft tissue resection and bone cutting, it may result in more blood loss. Accordingly, the risk of complications also increases after RKA.

Surgeons find it challenging to perform RKA as it is a major surgery with the potential to cause massive blood loss perioperatively. Several studies have reported the hematologic outcomes of TKA and RKA [18]. Prased et al. described that the transfusion rate of RKA was higher than that of TKA. However, to the best of our knowledge, the ideal Hb level for PJI to maintain good healing is not yet established. Thus, the present study was carried out to address the lack of knowledge of whether Hb level affects the curability of PJI. Results of this study showed no significant difference in the cement spacer retention period in terms of CRP levels and rate of cement spacer re-implantation.

Blood transfusions for perioperative blood loss in TKA or RKA may have fatal complications. Thus, blood transfusion is not a risk-free intervention. Blood transfusion can cause various complications, such as transfusion-transmitted infections, increasing length of hospital stay, risk for re-infections, respiratory infections, surgical wound infections, and systemic complications such as allergic reactions, graft-versus-host disease, transfusion-associated circulatory overload, and transfusion-related acute lung injury [19,20]. Furthermore, transfusion is associated with longer hospitalization stay and an increased hospital cost [21].

In addition to perioperative blood loss, preoperative anemia is often reported in patients who underwent TKA and RKA. In patients who underwent total hip or knee arthroplasty and hip fracture surgery, preoperative anemia was highly prevalent, which accounted for 24% to 44% of the patients [22] because most of these patients were women and had advanced age. In some studies, preoperative anemia is the strongest predictor of transfusion management [23]. The clinical application guidelines of the American Association of Blood Banks suggest that orthopedic surgeons should follow the transfusion standard of Hb < 8 g/dL [24]. A meta-analysis revealed that restrictive transfusion strategy results in a 43% decrease in transfusion rates, which was not related with fatal complications [25].

A 1 mg/dL decrease in Hb level is linearly associated with poorer outcomes. Preoperative anemia is associated with poorer surgical outcomes, and a 10 g/L decline in Hb level is linearly associated with an increased perioperative risk of 40% [26]. Hb level of 10 mg/dL is considered a critical level during surgery. There are practical guidelines that have been passed from one surgical resident to another even without confirmation by evidence-based research. The rule of 10 is also easy to remember and a practical guide [27].

In the last decade, we have utilized tranexamic acid (TXA) more frequently. Many studies have presented some strategies for management of blood loss perioperatively. TXA usage plays an important role. Many studies have shown that TXA can reduce blood loss and requirements for transfusion [28].

Study limitations

This study has several limitations, including the small number of patients and its retrospective design. Owing to the retrospective nature of the study, we cannot prove the causality between Hb levels and its effects on curability of PJI. The small numbers of patients confine the statistical analysis to a descriptive level. Further studies should include randomized controlled trials, and the sample size should be large enough to enable statistical analysis.

## Conclusions

In RKA, low Hb level was not a negative factor for the curability of infection. Thus, blood transfusions to increase Hb levels are not mandatory and can be avoided as they are associated with a high level of complications.
